# Preliminary study of a new magnetic compression technique for circumcision in dogs: An experimental animal model

**DOI:** 10.1016/j.heliyon.2024.e35646

**Published:** 2024-08-05

**Authors:** Miaomiao Zhang, Linxin Shen, Kaihua Xue, Aihua Shi, Yanfeng Gao, Yi Lyu, Xiaopeng Yan

**Affiliations:** a Department of Hepatobiliary Surgery, The First Affiliated Hospital of Xi'an Jiaotong University, Xi'an, 710061, China; b Shaanxi Provincial Key Laboratory of Magnetic Medicine, The First Affiliated Hospital of Xi'an Jiaotong University, Xi'an, 710061, China; c Zonglian College, Xi'an Jiaotong University, Xi'an, 710061, China; d Department of Urology, The First Affiliated Hospital of Xi'an Jiaotong University, Xi'an 710061, China; e National Local Joint Engineering Research Center for Precision Surgery & Regenerative Medicine, The First Affiliated Hospital of Xi'an Jiaotong University, Xi'an, 710061, China; f Department of Anesthesiology, The First Affiliated Hospital of Xi'an Jiaotong University, Xi'an, 710061, China

**Keywords:** Magnetic compression technique, Magnetosurgery, Beagle dogs, Circumcision, Redundant prepuce

## Abstract

**Introduction:**

Traditional/ritual/medical circumcision can be associated with considerable intraoperative blood loss and a prolonged postoperative healing course. This study investigated the feasibility of the magnetic compression technique (MCT) for circumcision in beagle dogs.

**Methods:**

A set of magnetic rings including a daughter magnetic ring (DMR) and a parent magnetic ring (PMR) were designed for circumcision. In eight beagle dogs as the animal model, the DMR was placed between the penis and the foreskin through the glans, and then the PMR was placed outside the penis. The DMR and PMR automatically attracted together to compress the foreskin. The necrosis of the prepuce of the anterior penis was observed daily. The operation time and time to magnetic ring shedding were recorded. Healing of the foreskin stump was visually observed.

**Results:**

The magnetic rings were successfully installed in all eight dogs, and the operation process was without complication. The average operation time was 3.13 ± 0.92 min (range, 2–4.5 min). Postoperative X-rays showed good attraction of the magnetic rings. Daily post-operative observation showed progressive ischemic necrosis of the anterior foreskin and mild edema of the proximal foreskin. The dogs were generally in good condition and urinated freely. The magnetic rings fell off spontaneously 8–12 days after the operation, and the stump of the foreskin healed well.

**Conclusion:**

The MCT may be a new approach for circumcision in a canine model, which suggests its potential for use in humans.

## 1 Introduction

Redundant prepuce is a frequent andrological condition among males worldwide [1]. Redundant prepuce can cause accumulation of smegma and repeated inflammatory stimulation, which has been shown to increase the incidence of penile cancer [2]. In addition, redundant prepuce increases the incidence of vaginitis, urinary tract infections, and even cervical cancer in sexual partners as well as the acquisition of some sexually transmitted diseases [3]. Some men with excessively long foreskin also experience premature ejaculation, which reduces the quality of sexual life for them and their partner [4,5]. Circumcision involves removal of the preputial tissue without causing damage to the glans penis. A study estimated that 37–39 % of men globally are circumcised [6]. Circumcision is even recognized as an ideal “surgical vaccine” against HIV infection [7,8]. In some faiths, circumcision is a religious obligation, and it is practiced on a large scale particularly in the Muslim community [1,9].

Due to a long operation duration, great intraoperative blood loss, and a prolonged postoperative healing course, traditional circumcision has lower acceptance among patients or their guardians. In recent years, disposable circumcision device offering the advantages of incisions with a favorable appearance, less bleeding, and short operation time have been widely used, gradually replacing traditional circumcision [10]. However, with their popularity in the clinic, some problems have also been identified.

The magnetic compression technique (MCT) is one of the most important clinical techniques in magnetic surgery [11]. In 1978, Obora reported the use of microvascular magnetic anastomosis in an animal experimental study [12]. Subsequently, the basic research and clinical application of the MCT were extended to many surgical procedures, including portacaval shunting [13,14], cystostomy [15], ureterovesical anastomosis [16], digestive tract anastomosis [[17], [18], [19]], and animal model preparation of tracheoesophageal fistula [20]. Zhang et al. established the first stage of magnetic anastomosis of the digestive tract using the model of colonic magnetic anastomosis in rats, and this procedure is now often called Yan-Zhang's staging [21]. Based on previous studies of the MCT, we hypothesize that the suture of the residual end after circumcision can be regarded as anastomosis of the inner and outer layers of the foreskin, and this anastomosis can be achieved using the MCT. This is the theoretical basis for the present study. Here, we designed and validated the feasibility of using a pair of magnetic rings for complete circumcision in beagle dogs as an animal model. This study has the potential to provide a new surgical approach to circumcision.

## 2 Materials and methods

### 2.1 Yan-Zhang's circumcision magnetic ring

**Fig. 1:**
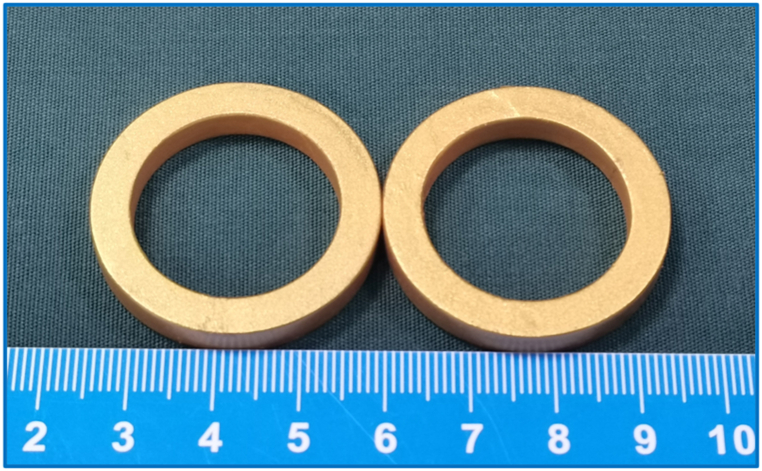
Yan-Zhang's circumcision magnetic ring.

**Fig. 2:**
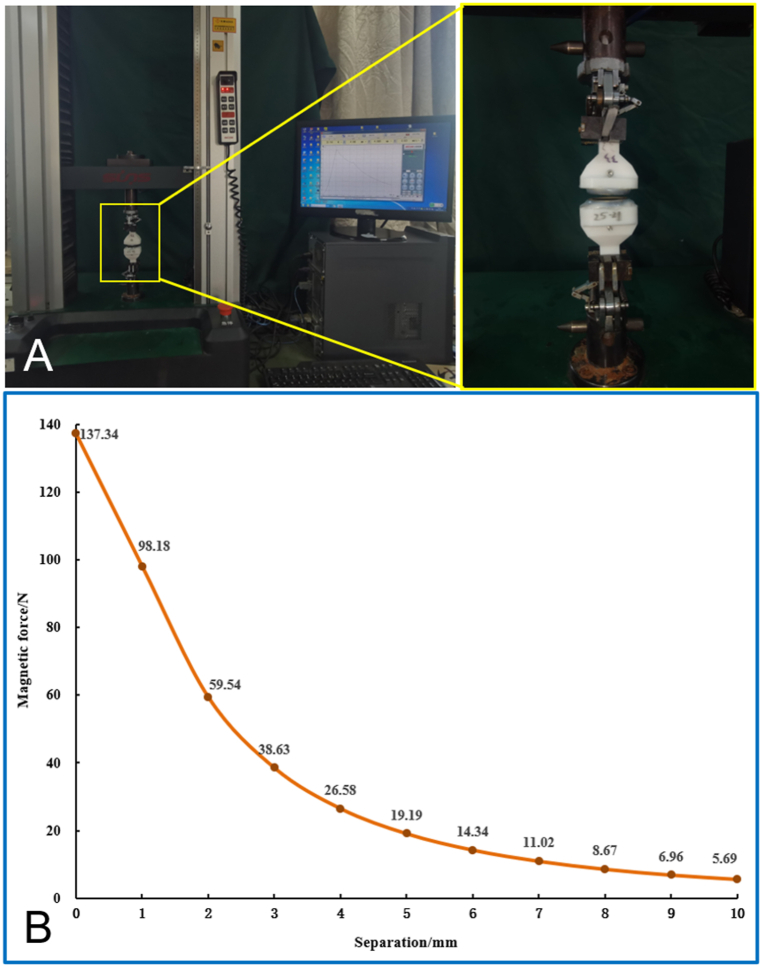
**Magnetism of the daughter and parent magnetic rings. A** Magnetism test equipment. **B** Magnetic force-displacement curve for the daughter and parent magnetic rings.

The Yan-Zhang's circumcision magnetic ring (Yan-Zhang's CMR) consists of two parts: a daughter magnetic ring (DMR) and a parent magnetic ring (PMR). The size and shape of the daughter magnetic ring used in this experiment were the same as that of the parent magnetic ring. Both rings were prepared by N52 sintering NdFeB followed by modification with titanium nitride. The outer diameter, inner diameter, and height of the magnetic rings were 35, 25, and 5 mm, respectively. The Yan-Zhang's CMR is shown in Fig. 1. For widespread application, magnetic rings of different sizes can be designed to match the thickness of the penis. The Yan-Zhang's CMR used in this study was designed by the authors (Xiaopeng Yan and Miaomiao Zhang) and manufactured by Jinshan Electronic Appliances, Ltd. (Xi'an, China). The mass of a magnetic ring was 16.8 g, and the magnetic induction intensity of the working surface was 3730 Gs. The magnetic force between the two rings was 137.34 N at zero distance. The magnetic force curve is shown in Fig. 2.

### 2.2 Animals

Eight male beagle dogs, aged 1–6 years (median: 3 years), weighing 12–15 kg (median: 12.5 kg), were obtained from the Laboratory Animal Center, Xi'an Jiaotong University, Xi'an, China. The study was conducted in strict accordance with the recommendations of the Xi'an Jiaotong University Medical Center Guide for the Care Use of Laboratory Animals. The animals were acclimatized to laboratory conditions (23 °C, 12h/12h light/dark, 50 % humidity, *ad libitum* access to food and water) for one week prior to commencing the experiments. The study protocol was approved by the Committee for Ethics of Animal Experiments at Xi'an Jiaotong University (Permit Number: XJTUAE2023-2206).

### 2.3 Study design

**Fig. 3:**
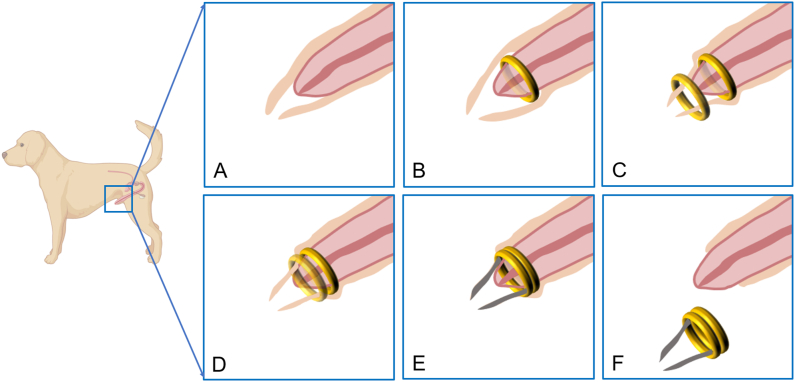
**Schematic representation of the surgical procedure using Yan-Zhang's circumcision magnetic ring. A** The foreskin is long and wraps around the head of the penis. **B** The daughter magnetic ring is inserted through the balanus. **C** The parent magnetic ring is applied. **D** The parent magnetic ring and daughter magnetic ring attract together. **E** After a few days of magnetic ring compression, the overlong foreskin becomes ischemic and necrotic. **F** The magnetic rings fall off along with the ischemic-necrotic foreskin.

Because this was an exploratory experimental study, all eight beagle dogs were included in the experimental investigation, and none served as controls. The study is reported in accordance with ARRIVE guidelines. For the experimental procedure, the DMR was placed between the penis and the foreskin through the glans, and then the PMR was placed outside the penis. The DMR and PMR automatically attracted together to compress the foreskin (Fig. 3A–D). Over the course of a few days, the compressed foreskin would experience annular ischemic necrosis and fall off, after which the circumcision procedure was complete (Fig. 3E and F).

### 2.4 Surgical procedure

**Fig. 4:**
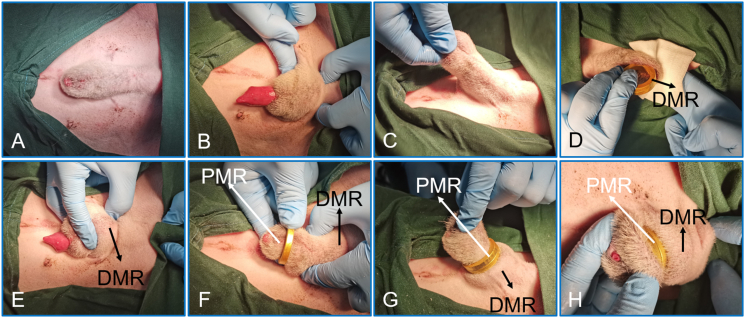
**Procedure of the MCT for circumcision. A** The penis is sterilized and covered with a sterile towel. **B** and **C** The length of the circumcision is determined by turning and pulling the foreskin. **D** The daughter magnetic ring is inserted. **E** The daughter magnetic ring is adjusted to ensure proper positioning. **F** The parent magnetic ring is applied. **G** The daughter magnetic ring and parent magnetic ring automatically attract. **H** The urethral opening is observed by pulling the magnetic rings and foreskin.

Adaptive feeding was performed for the beagle dogs for 1 week after purchase. Food and water were prohibited 4 h preoperatively, and 3 % pentobarbital sodium (1 ml/kg) was intravenously injected [[14], [18], [19]]. After satisfactory anesthesia (spontaneous breathing is stable, limbs are relaxed and unresponsive to painful stimuli), the beagle dogs were placed in the supine position, and the hair on the lower abdomen and penis was removed. The penis was sterilized and covered with a sterile towel (Fig. 4A). The penis and the inner side of the prepuce were coated with tetracaine paste. The length of the circumcision was determined by turning and pulling the foreskin (Fig. 4B-C). The DMR was placed into the foreskin (Fig. 4D), and the head of the penis was passed through the DMR. The position of the DMR was adjusted so that it was located between the penis and the foreskin (Fig. 4E). The PMR was placed outside the foreskin and pushed towards the base of the penis (Fig. 4F). The PMR and DMR automatically attracted each other (Fig. 4G). At this time, the long foreskin of the head of the penis was compressed by the DMR and PMR. The foreskin and magnetic rings were pulled together again to ensure that the penis was properly relaxed and the urethral opening was visible, to ensure normal urination was possible (Fig. 4H). With the passage of time, the prepuce at the head of the penis underwent the pathological change process of ischemia–necrosis and eventually fell off along with the magnetic rings, and the proximal prepuce that had been compressed by the magnetic rings was allowed to heal itself.

### 2.5 Calculation of operation time

The operation time, defined as the time from the completion of disinfection until the DMR and PMR attracted together, was recorded for all beagle dogs.

### 2.6 Postoperative care

Immediately postoperatively, a plain lower abdomen X-ray of the beagle dogs was taken to confirm the position and status of the DMR and PMR. After recovery from anesthesia, all beagle dogs were given food and water freely in a single cage. During the first 5 days after operation, the animals received an intramuscular pethidine (1 mg/kg) injection every 12 h. Antibiotics are not used in the perioperative period. The penis was disinfected with Aner iodine mucosa disinfectant (Shanghai Likang Disinfectant Hi-Tech Co., Ltd., Shanghai, China) daily.

### 2.7 Postoperative observation

The progression of foreskin necrosis was observed daily, and the urination of the dogs was observed. The time until foreskin and magnetic ring shedding was recorded. Postoperative complications including bleeding, infection, and edema were recorded. Healing of the stump of the foreskin was observed by naked eye after the magnetic rings had fallen off.

### 2.8 Statistical analysis

SPSS v22.0 was used for data analysis. Quantitative data are expressed as mean ± standard deviation (SD).

## 3 Results

### 3.1 Operation time

The MCT for circumcision was completed in all eight beagle dogs, with a mean operation time of 3.13 ± 0.92 min (range, 2–4.5 min).

### 3.2 Survival rate and postoperative complications

**Fig. 5:**
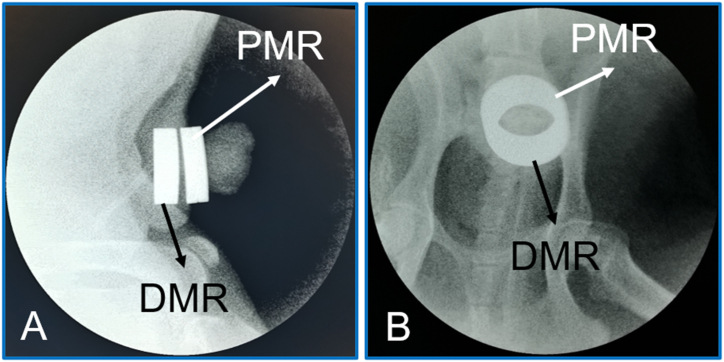
**X-ray shows that the daughter and parent magnet rings are positioned as designed. A** Lateral X-ray. **B** Anteroposterior X-ray.

**Fig. 6:**
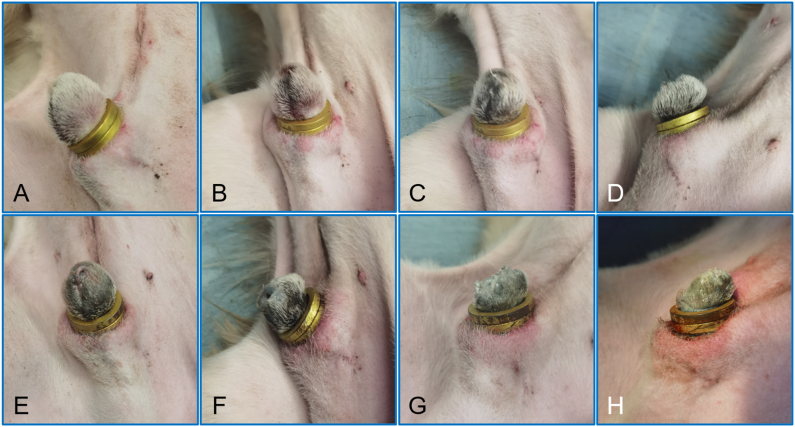
**Process of necrosis and shedding of the foreskin after operation. A** Purple foreskin could be seen 24 h after surgery. **B** The color change of the foreskin was more severe at 48 h after surgery. **C** Black foreskin was observed 72 h after operation, and mild edema of the foreskin near the magnetic rings was observed. **D** and **E** Anterior prepuce necrosis and penile edema continued with the same appearance on the 4th and 5th postoperative days. **F** and **G** Further necrosis of the anterior prepuce with partial tissue decay was observed on the 6th and 7th days. **H** Part of the tissue between the daughter magnetic ring and the parent magnetic ring was necrotic and detached on the eighth day after surgery.

Postoperative X-ray examination indicated that placement of the DMR and PMR was achieved according to the procedure design (Fig. 5A-B). All beagle dogs survived postoperatively. No intraoperative or postoperative complications were observed, including bleeding and infection. Free urination of the experimental dogs was not affected. Mild edema was observed in the proximal prepuce near the magnetic rings starting on the second day after surgery, reaching a peak on the fifth day, and then gradually subsiding (Fig. 6).

### 3.3 Magnet ring discharge time

The mean time required for the magnetic rings to be expelled from the beagle dogs’ penis was 9.63 ± 1.41 days (range, 8–12 days) after placement.

### 3.4 Gross appearance of the stump of foreskin

**Fig. 7:**
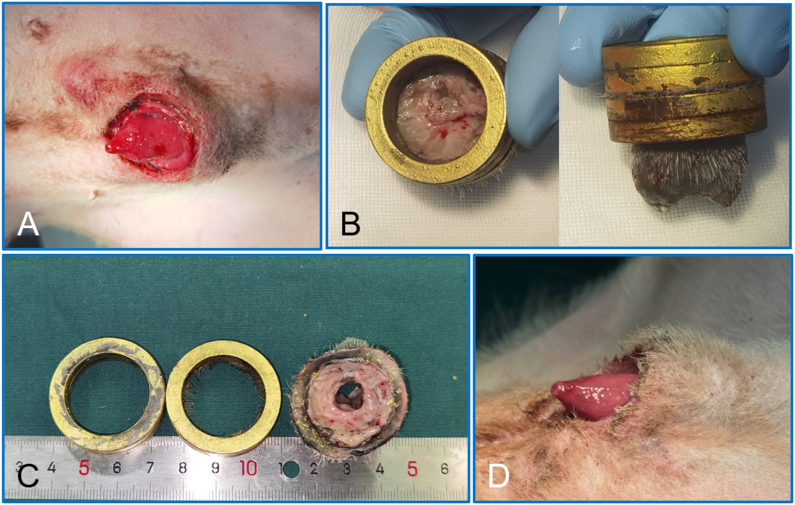
**The parent and daughter magnetic rings fell off, and the circumcision was completed. A** The penis after the magnetic rings fell off. **B** The magnetic rings after they fell off the penis. **C** Necrotic prepuce tissue was observed upon separation of the daughter magnetic ring from the parent magnetic ring. **D** The stump of the foreskin observed on the fourth day after the magnetic rings had detached.

After the magnetic rings had fallen off, good healing was observed by visual inspection. There is no bleeding, infection, discharge or swelling could be seen at the stump of the foreskin (Fig. 7).

## 4 Discussion

In this study, we successfully applied the MCT to circumcision. To our knowledge, this is the first time use of the MCT for circumcision has been reported. During the operation, the magnetic rings were applied without any injury to the penis or foreskin. No significant complications occurred during or after the operation, and the stump of the foreskin healed well after automatic detachment of the magnetic rings. We found that the healing of the remnant of the foreskin was very similar to the healing of the tissue after magnet shedding during magnetic compression gastrostomy and magnetic compression cystostomy[22,23]. The presented experiment demonstrates the feasibility of using the MCT for circumcision. Several aspects of the study should be considered.

In this study, we used beagle dogs as the animal model. Although the external genitalia of beagles are quite different from those of humans, we have not yet found a better animal with congenital hyper foreskin than beagle dogs. Unlike humans, the beagle dog's foreskin is a continuation of the skin of the lower abdomen. Thus, the foreskin tissue is thicker, and there is no obvious frenulum structure. Furthermore, beagle dogs do not have a human-like coronal groove at the proximal end of the glans. Based on the above characteristics, we placed the DMR at the distal end of the bulbourethra gland in the dogs, and the PMR at the junction of the free segment of the foreskin and the skin of the abdominal wall, which is equivalent to the coronal groove of the human penis.

In terms of magnetic ring design, we should fully consider the following points: (1) the dog's penis has a penis bone, so when the DMR was set on the penis, a certain amount of space must be allowed not only to prevent the ring from being too tight and causing ischemic necrosis of the penis, but also to avoid dog penile erection caused by locking, which is the basic requirement for the inner diameter of the magnetic ring. This point is consistent with the design requirements of the ShangRing, and the size of the ring should be selected according to the outside diameter of the penis when in use[24]. (2) As previously mentioned, the beagle dog's foreskin tissue is thicker, so when the PMR is loaded on to the penis, the structure differs from that inside the DMR. The DMR is around only the penis, whereas the PMR surrounds the penis and foreskin. Accordingly, it is necessary to ensure the tissue is not compressed too tightly by the PMR, which could affect the ability to urinate. (3) The DMR is located in the gap between the penis and the foreskin, and the size of this gap is the basis for the design of the outer diameter of the DMR. (4) In the initial design of the magnetic rings, we adopted a scheme with a height of 5 mm, but during the operation, we observed that when a single magnetic ring was used as the PMR or a DMR, the magnetic force between them was weak. Thus, we adopted the scheme of two magnetic rings superimposed as the PMR and DMR, and finally achieved success. In magnetic compression anastomosis, the optimal magnetic force is a critical consideration. Researchers have studied the optimal magnetic force for intestinal anastomosis and biliointestinal anastomosis previously[25,26]. Further studies are needed to determine the optimal magnetic force for circumcision.

While the study demonstrated the effectiveness of the magnetic compression technique for circumcision, it is important to note some limitations. The experiment only involved eight beagles, suggesting a need for a larger sample size to ensure more reliable results. Additionally, the use of magnetic rings with a fixed outer diameter of 35 mm limited the scope of experimental data. Future studies should explore the use of magnetic rings with varying sizes on animals of different weights to gather more comprehensive information. Moving forward, improvements in the magnetic ring structure will be a focus in upcoming research programs. Once clinical ethical approval is obtained, suitable patients will be selected for future trials.

## 5 Conclusion

As the first exploratory experiment to apply the MCT to circumcision, despite limitations regarding the number of animals and model selection, the experimental results offer significant promise. The simple surgical procedure, negligible postoperative complications, and good healing effect achieved suggest that this technique has the potential to be used in humans.

## Funding

This study was supported by the 10.13039/501100017592Key Research & Development Program of Shaanxi Province of China (2024SF-YBXM-447), the Institutional Foundation of The First Affiliated Hospital of 10.13039/501100016347Xi'an Jiaotong University (No. 2022MS-07), the 10.13039/501100012226Fundamental Research Funds for the Central Universities (No. xzy022023068) and Heye Health Science and Technology Foundation- Magnetic Surgical Technique and the Basic Research (No. HX202197).

## ARRIVE guidelines statement

The authors have read the ARRIVE guidelines, and the manuscript was prepared and revised according to the ARRIVE guidelines.

## Ethic statement

This study was reviewed and approved by the Committee for Ethics of Animal Experiments at Xi'an Jiaotong University with the approval number: [XJTUAE2023-2206], dated [November 13, 2023].

## Data availability statement

The data included in article as well as more detailed data can be obtained by E-mail to yanxiaopeng99@163.com.

## CRediT authorship contribution statement

**Miaomiao Zhang:** Writing – original draft, Project administration, Methodology, Investigation, Funding acquisition, Formal analysis, Data curation, Conceptualization. **Linxin Shen:** Methodology, Investigation, Formal analysis, Data curation. **Kaihua Xue:** Methodology, Investigation, Formal analysis, Data curation. **Aihua Shi:** Methodology, Investigation, Formal analysis. **Yanfeng Gao:** Methodology, Investigation. **Yi Lyu:** Writing – review & editing, Supervision, Project administration, Methodology, Funding acquisition, Conceptualization. **Xiaopeng Yan:** Writing – review & editing, Writing – original draft, Supervision, Project administration, Methodology, Investigation, Funding acquisition, Formal analysis, Data curation, Conceptualization.

## Declaration of competing interest

The authors declare that they have no known competing financial interests or personal relationships that could have appeared to influence the work reported in this paper.
